# PGC-1α Deficiency Causes Multi-System Energy Metabolic Derangements: Muscle Dysfunction, Abnormal Weight Control and Hepatic Steatosis

**DOI:** 10.1371/journal.pbio.0030101

**Published:** 2005-03-15

**Authors:** Teresa C Leone, John J Lehman, Brian N Finck, Paul J Schaeffer, Adam R Wende, Sihem Boudina, Michael Courtois, David F Wozniak, Nandakumar Sambandam, Carlos Bernal-Mizrachi, Zhouji Chen, John O. Holloszy, Denis M Medeiros, Robert E Schmidt, Jeffrey E Saffitz, E. Dale Abel, Clay F Semenkovich, Daniel P Kelly

**Affiliations:** **1**Center for Cardiovascular Research, Washington University School of MedicineSt Louis, MissouriUnited States of America; **2**Department of Medicine, Washington University School of MedicineSt Louis, MissouriUnited States of America; **3**Program in Human Molecular Biology and Genetics, Division of EndocrinologyMetabolism and Diabetes, University of Utah, Salt Lake City, UtahUnited States of America; **4**Department of Psychiatry, Washington University School of MedicineSt Louis, MissouriUnited States of America; **5**Department of Human Nutrition, Kansas State UniversityManhattan, KansasUnited States of America; **6**Department of Pathology, Washington University School of MedicineSt Louis, MissouriUnited States of America; **7**Department of Molecular Biology and Pharmacology, Washington University School of MedicineSt Louis, MissouriUnited States of America; **8**Department of Pediatrics, Washington University School of MedicineSt Louis, MissouriUnited States of America; University of CambridgeUnited Kingdom

## Abstract

The gene encoding the transcriptional coactivator peroxisome proliferator-activated receptor-γ coactivator-1α (PGC-1α) was targeted in mice. PGC-1α null (PGC-1α^−/−^) mice were viable. However, extensive phenotyping revealed multi-system abnormalities indicative of an abnormal energy metabolic phenotype. The postnatal growth of heart and slow-twitch skeletal muscle, organs with high mitochondrial energy demands, is blunted in PGC-1α^−/−^ mice. With age, the PGC-1α^−/−^ mice develop abnormally increased body fat, a phenotype that is more severe in females. Mitochondrial number and respiratory capacity is diminished in slow-twitch skeletal muscle of PGC-1α^−/−^ mice, leading to reduced muscle performance and exercise capacity. PGC-1α^−/−^ mice exhibit a modest diminution in cardiac function related largely to abnormal control of heart rate. The PGC-1α^−/−^ mice were unable to maintain core body temperature following exposure to cold, consistent with an altered thermogenic response. Following short-term starvation, PGC-1α^−/−^ mice develop hepatic steatosis due to a combination of reduced mitochondrial respiratory capacity and an increased expression of lipogenic genes. Surprisingly, PGC-1α^−/−^ mice were less susceptible to diet-induced insulin resistance than wild-type controls. Lastly, vacuolar lesions were detected in the central nervous system of PGC-1α^−/−^ mice. These results demonstrate that PGC-1α is necessary for appropriate adaptation to the metabolic and physiologic stressors of postnatal life.

## Abstract 2

Eliminating the activity of the gene PGC-1 α in mice reveals its role in post-natal metabolism and provides a link to obesity and some intriguing differences with another report of this knockout

## Introduction

Mitochondrial functional capacity is dynamically regulated to meet the diverse energy demands imposed on the mammalian organism following birth. Postnatal mitochondrial biogenesis involves multiple signaling and transcriptional regulatory pathways that control the coordinate expression of nuclear and mitochondrial genes involved in mitochondrial structure, metabolism, and proliferation [1]. Recent evidence points toward a transcriptional coactivator, peroxisome proliferator-activated receptor-γ (PPARγ) coactivator-1α (PGC-1α), as an integrator of the molecular regulatory circuitry involved in the transcriptional control of cellular energy metabolism, including mitochondrial function and biogenesis [1,2]. PGC-1α was discovered in a yeast two-hybrid screen for brown adipose-specific factors that interact with the adipogenic nuclear receptor PPARγ [2]. Subsequently, two additional PGC-1 family members were identified, PGC-1 related coactivator (PRC) [3] and PGC-1β [4,5]. PGC-1α serves as a direct transcriptional coactivator of nuclear and nonnuclear receptor transcription factors involved in cellular energy metabolism [6]. PGC-1α is distinct among most coactivators in that it exhibits a tissue-enriched expression pattern and is highly inducible by physiologic conditions known to increase the demand for mitochondrial ATP or heat production [2,6,7]. PGC-1α is enriched in brown adipose tissue (BAT), heart, slow-twitch skeletal muscle, and kidney—all tissues with high-capacity mitochondrial systems. The expression of the gene encoding PGC-1α is rapidly induced by cold exposure, short-term exercise, and fasting [2,8,9,10,11,12,13,14,15]. These latter observations suggest that PGC-1α is involved in the physiologic control of energy metabolism.

Several lines of evidence, based on the results of overexpression studies, indicate that PGC-1α is sufficient to promote mitochondrial biogenesis and regulate mitochondrial respiratory capacity. First, PGC-1α activates the transcription of mitochondrial uncoupling protein-1 (UCP-1) in BAT through interactions with the nuclear hormone receptors PPARγ and thyroid receptor [2]. Second, forced expression studies in adipogenic and myogenic mammalian cell lines demonstrated that PGC-1α activates mitochondrial biogenesis through a group of transcription factor targets including nuclear respiratory factors 1 and 2 (NRF-1 and -2) and mitochondrial transcription factor A (Tfam), key transcriptional regulators of mitochondrial DNA transcription and replication [8]. Third, studies in primary cardiac myocytes in culture and in the hearts of transgenic mice have demonstrated that overexpression of PGC-1α promotes mitochondrial biogenesis [10,16]. Lastly, forced expression of PGC-1α in skeletal muscle of transgenic mice triggers mitochondrial proliferation and the formation of mitochondrial-rich type I, oxidative (“slow-twitch”) muscle fibers [17]. Collectively, these results indicate that PGC-1α is sufficient to drive mitochondrial biogenesis.

Recent evidence also implicates PGC-1α in the homeostatic control of systemic energy metabolism. PGC-1α has been shown to regulate several key hepatic gluconeogenic genes [18,19,20,21]. Recent studies have also shown altered expression of PGC-1α and downstream mitochondrial target pathways in skeletal muscle of humans with insulin resistance and diabetes [22,23,24]. In addition, single nucleotide polymorphisms within the human *PGC-1α* gene have been shown to be associated with obesity, hypertension, and diabetes [25,26,27,28,29,30].

The gain-of-function studies described to date provide compelling evidence that PGC-1α is capable of regulating postnatal energy metabolism. However, the *necessity* of PGC-1α for energy metabolic homeostasis, mitochondrial biogenesis, development, and growth can only be addressed using loss-of-function strategies. To this end, we have established and characterized mice with targeted deletion of the *PGC-1α* gene. Our studies of PGC-1α^−/−^ mice demonstrate that PGC-1α is not absolutely required for prenatal viability including mitochondrial biogenesis. However, our findings indicate that the coactivator PGC-1α serves a critical role in the normal metabolic function of multiple organs and for appropriate adaptation to physiologic stress during postnatal life.

## Results

### Disruption of the PGC-1α Gene in Mice

**Figure 1:**
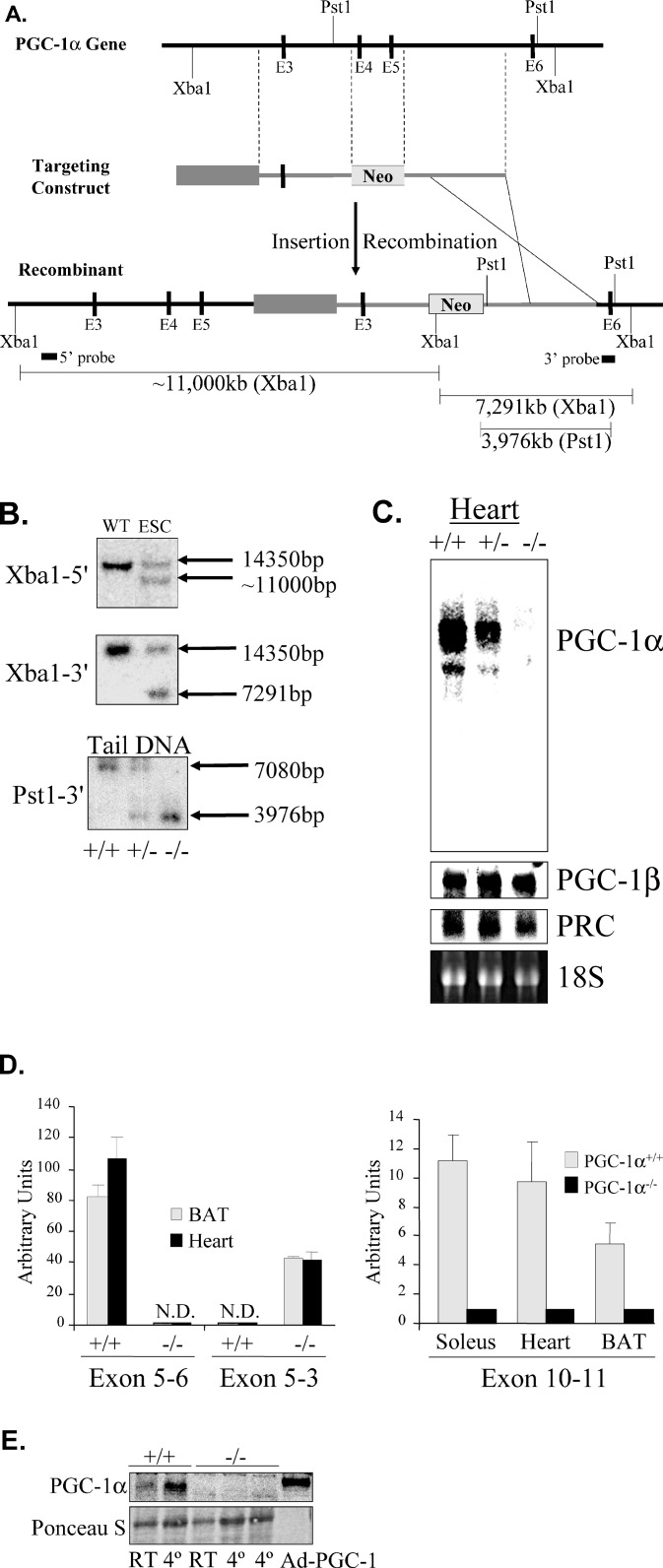
Deletion of the PGC-1α Gene (A) Schematic of the gene targeting strategy. A region of the murine *PGC-1α* gene containing exons 3–6 is shown schematically at the top. Relevant restriction endonuclease sites are also shown. The targeting construct containing a neomycin (Neo) cassette is shown below the *PGC-1α* gene with dashed lines indicating the regions targeted for homologous recombination. Homologous recombination between the 3′ end of the targeting vector and the *PGC-1α* gene is indicated by the solid lines. The targeting construct inserted into the *PGC-1α* gene resulting in a duplication of exon 3 and incorporation of the targeting construct DNA into the final recombinant as shown. Probes used for the Southern blot studies and relevant restriction fragments predicted by digestion of the recombinant are also shown. (B) Southern blot analysis of embryonic stem cells (ESC) (digested with Xba1) and tail DNA (digested with Pst1) is shown. The blots were hybridized with the probes shown in Figure 1A. Results for PGC-1α^+/+^ (+/+), PGC-1α^+/−^ (+/−), and PGC-1α^−/−^ (−/−) genotypes are shown as denoted at the bottom. (C) Northern blot analysis using RNA isolated from the hearts of the three relevant PGC-1α genotypes (as in Figure 1B) is shown using PGC-1α cDNA as a probe. In addition, PGC-1β and PRC cDNA probes were used as shown. Ethidium bromide staining of 18S ribosomal RNA is shown at the bottom. (D) Quantitative real time RT-PCR (Sybr green) was used to detect PGC-1α transcripts using primer sets crossing different exon borders as denoted. The exon 5–3 primer set detects only the mutant transcript (−/−), whereas the exon 5–6 primer set detects only the WT transcript. The values represent arbitrary units for RNA isolated from the tissues shown for the three amplicons comparing PGC-1α^+/+^ and PGC-1α^−/−^. N.D. = not detectable. The exon 10–11 amplicon was evaluated to assess levels of mutant versus WT transcripts. The values represent arbitrary units normalized to actin control. (E) Western blot analysis using whole-cell protein extracts prepared from BAT under basal conditions and following exposure to 4 °C for 8 h. The signal shown was obtained with polyclonal anti-PGC-1α antibody [10]. An epitope-tagged PGC-1α, overexpressed in neonatal cardiac myocytes using an adenoviral vector (Ad-PGC-1α), is shown as a positive control. The Ponceau S stain of the protein gel is shown at the bottom.

A neomycin-based gene targeting vector was generated to delete exons 4 and 5 of the murine *PGC-1α* gene. The targeting event resulted in a 3′ homologous recombination with insertion of the remainder of the construct (Figure 1A). The insertion/recombination event was confirmed by Southern blotting and DNA sequencing. The insertion caused an exon 3 duplication between exons 5 and 6 that creates a coding region frameshift resulting in a premature termination at amino acid 255. Germline transmission of the mutant allele was confirmed using Southern blotting (Figure 1B) and PCR (unpublished data). The *PGC-1α* gene disruption resulted in an unstable transcript that could not be detected by RNA blot analysis in heart and other tissues in PGC-1α^−/−^ mice (Figure 1C and unpublished data). Quantitative RT-PCR was utilized to further evaluate the efficacy of the gene targeting. For these studies, PCR primers were designed to amplify a region of the *PGC-1α* gene transcript containing the exon 5–6 border (predicted to be absent in PGC-1α^−/−^ mice) or the exon 5–3 border (predicted to be present only in the PGC-1α^−/−^ mice). The exon 5–6 amplicon was detected in heart and BAT of wild-type (WT) but not PGC-1α^−/−^ mice (Figure 1D). Conversely, the exon 5–3 product was present only in PGC-1α^−/−^ mice (Figure 1D). An exon 10–11 border amplicon (predicted to be present in both genotypes) was detected in WT and PGC-1α^−/−^ mice, but was greatly diminished in the PGC-1α^−/−^ mice, indicating that the mutant transcript is unstable. PGC-1α protein was not detected in whole cell (Figure 1E) or nuclear protein extracts (unpublished data) isolated from BAT of PGC-1α^−/−^ mice under basal conditions or in response to cold exposure, a condition known to markedly induce the expression of PGC-1α in BAT. Smaller mutant PGC-1α proteins were also not detected by Western blot analysis (unpublished data). Lastly, expression of the genes encoding the other known PGC-1 family members, PGC-1β and PRC, was not significantly altered in heart of PGC-1α^−/−^ mice (Figure 1C). Taken together, these results support the conclusion that the gene targeting event resulted in a PGC-1α null allele.

### General Characteristics of the PGC-1α^−/−^ Mice: Age- and Sex-Dependent Obesity

**Figure 2:**
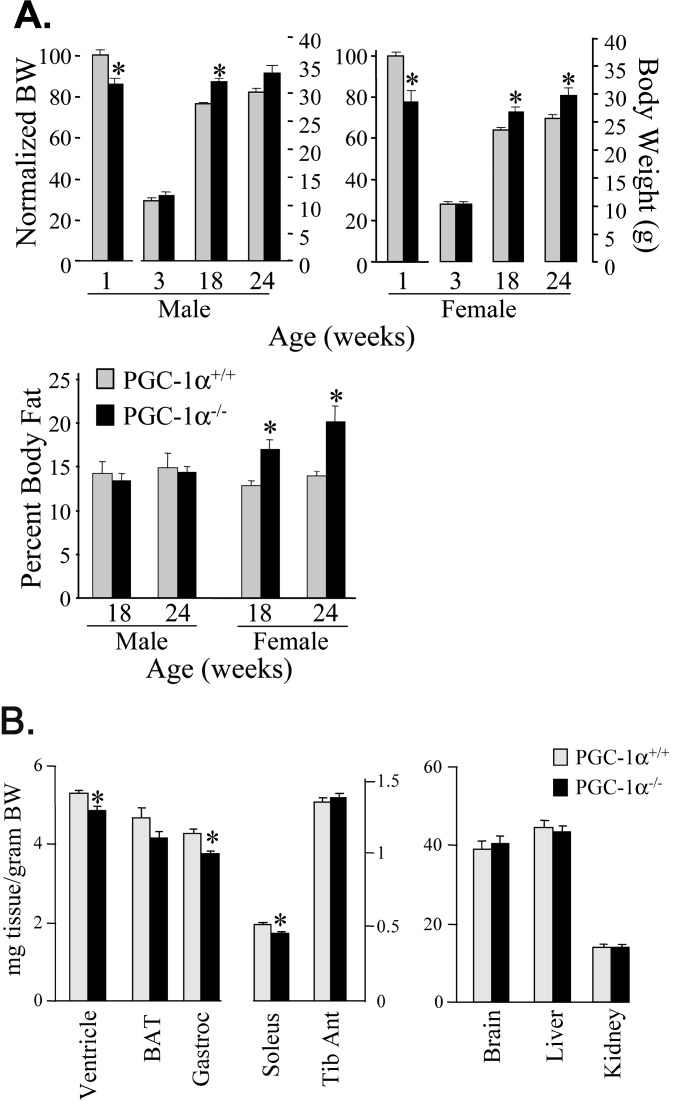
Evidence for Tissue-Specific Growth Abnormalities and Mild Sex-Limited, Age-Dependent Obesity in PGC-1α^−/−^ Mice (A) The bars represent total body weight for the ages indicated for male (left graph) and female (center graph) PGC-1α^+/+^ and PGC-1α^−/−^ mice. The body weight (BW) of the 1-wk-old PGC-1α^−/−^ mice was normalized to that of PGC-1α^+/+^ littermates, which was assigned a value of 100 (left axis). For the 3-, 18-, and 24-wk time points, absolute weights of PGC-1α^−/−^ mice were compared to age-matched controls (right axis). Percent fat as determined by DEXA scanning for PGC-1α^+/+^ and PGC-1α^−/−^ mice (right graph). The results represent *n* = 4 (males) and *n* ≥ 11 (females) for each genotype at 24 wk. * *p* < 0.05 compared to corresponding PGC-1α^+/+^ mice. (B) The bars represent organ weights corrected to body weight for 3-wk-old male and female PGC-1α^+/+^ and PGC-1α^−/−^ mice. The error bars represent ± SEM. Results represent *n* ≥ 14 for each group. * *p* < 0.05 compared to corresponding PGC-1α^+/+^ mice.

Heterozygous (PGC-1α^+/−^) mice were bred to generate PGC-1α^−/−^ offspring. The observed genotype ratios of the offspring were consistent with the expected Mendelian ratios (unpublished data). Unexpected deaths of the offspring were not observed, and PGC-1α^+/−^ and PGC-1α^−/−^ offspring appeared normal. Total body weights obtained 1 wk after birth revealed a 15%–20% reduction in total body mass for male and female PGC-1α^−/−^ mice relative to sex-matched PGC-1α^+/+^ littermates (Figure 2). The weight decrement between PGC-1α^−/−^ and PGC-1α^+/+^ littermates disappeared by 3 wk of age (Figure 2A). At 18 wk of age, body weight was modestly but significantly greater in male and female PGC-1α^−/−^ mice compared to sex-matched PGC-1α^+/+^ controls (Figure 2A). This weight difference was also significant for female PGC-1α^−/−^ mice at 24 wk of age (Figure 2A). The abnormal weight gain in PGC-1α^−/−^ mice was not associated with differences in food intake (unpublished data) or alterations in general activity as monitored for 48 h (Figure S1). Percent body fat, as determined by dual-energy X-ray absorption (DEXA), was greater in 18- and 24-wk-old female PGC-1α^−/−^ mice compared to age-matched female PGC-1α^+/+^ counterparts, indicating that the body weight difference was due, at least in part, to increased body fat (Figure 2A). Lean mass was not significantly different between the genotypes (unpublished data). Although DEXA did not detect excess body fat in male PGC-1α^−/−^ mice at 18 or 24 wk of age, older male mutant mice (over 7 mo of age) accumulated more body fat than male WT controls (Figure 2A and unpublished data).

Individual organ weights were assessed, given the importance of mitochondrial energy metabolism for postnatal growth in certain organs. The weights of heart and slow-twitch fiber-enriched skeletal muscles, including gastrocnemius and soleus, but not the less oxidative tibialis anterior, were significantly lower in male and female PGC-1α^−/−^ mice compared with age and sex-matched PGC-1α^+/+^ controls at 3 and 8 wk of age (Figure 2B and unpublished data). In contrast, the weights of brain, liver, kidney, and BAT were not significantly different between the genotypes at the 3-wk time point (Figure 2B). Thus, certain tissues with high mitochondrial energy requirements, such as heart and slow-twitch skeletal muscle, exhibit modest growth defects in PGC-1α^−/−^ mice.

### Abnormal Muscle Mitochondrial Phenotype in PGC-1α^−/−^ Mice

**Figure 3:**
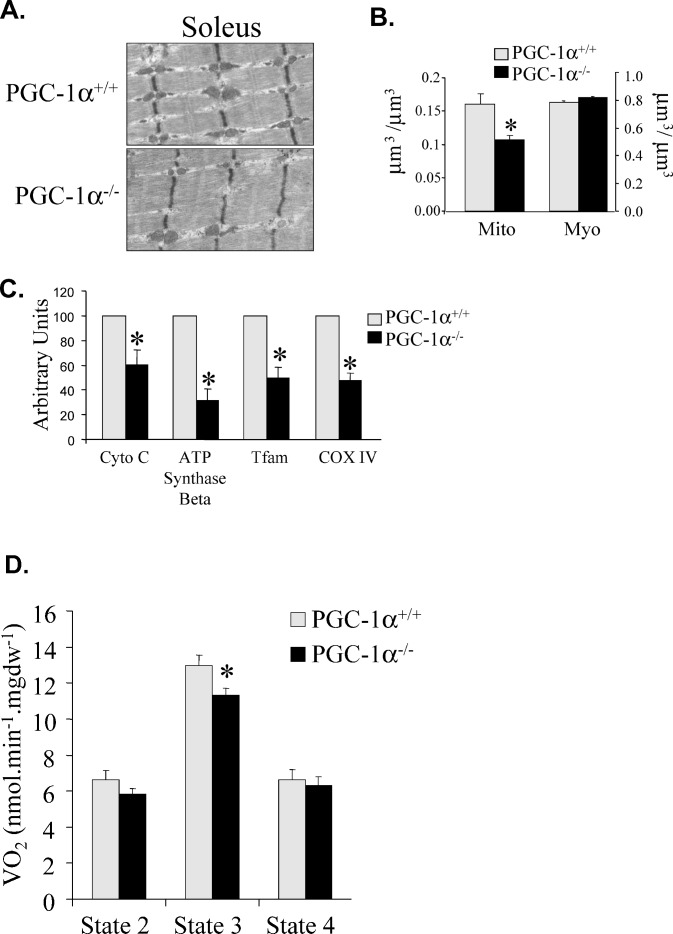
Abnormal Mitochondrial Phenotype in Slow-Twitch Skeletal Muscle of PGC-1α^−/−^ Mice (A) Representative electron micrograph of soleus muscle from 1-mo-old female PGC-1α^+/+^ and PGC-1α^−/−^ mice. (B) Quantitative morphometric measurements of the cellular volume density for the mitochondrial (Mito) and myofibrillar (Myo) fractions based on analysis of electron micrographs (three sections from three animals per group). The bars represent mean ± SEM. * *p* < 0.05 compared to corresponding PGC-1α^+/+^ values. (C) Gene expression data. The results of real-time PCR analysis of nuclear and mitochondrial genes involved in various components of mitochondrial metabolism and mitochondrial biogenesis: cytochrome C (Cyto c), ATP synthase β, Tfam, and cytochrome oxidase IV (COX IV). Eight littermate pairs were used for analysis at 1–2 mo of age and normalized to the WT value, which was assigned a value of 100, in each case. * *p* < 0.05 compared to corresponding PGC-1α^+/+^ values. (D) Mitochondrial respiration rates as determined by oxygen consumption (VO_2_) performed on saponin-permeabilized muscle strips prepared from soleus of PGC-1α^+/+^ and PGC-1α^−/−^ mice (as described in Materials and Methods). The results are based on six female animals in each group, using succinate as a substrate in the presence of rotenone. Mean values (± SEM) are shown for state 2 (basal), state 3 (ADP-stimulated), and state 4 respiration (presence of oligomycin).

General histologic analyses were performed to begin to evaluate the mild growth defect found in postnatal heart and skeletal muscle of the PGC-1α^−/−^ mice. There were no obvious abnormalities in cellularity, cell size, or extracellular matrix in the tissues of 1–2-mo-old PGC-1α^−/−^ mice (unpublished data). Given the important role of PGC-1α in mitochondrial function and biogenesis, we examined mitochondrial ultrastructure in the relevant tissues. Electron microscopic analysis revealed fewer and smaller mitochondria in soleus muscle of PGC-1α^−/−^ mice compared to sex- and age- matched PGC-1α^+/+^ controls (Figure 3A). Quantitative morphometry of the electron micrographs confirmed that the cellular volume density of soleus mitochondria was significantly lower in PGC-1α^−/−^ mice compared to PGC-1α^+/+^ controls independent of changes in the myofibrillar component (Figure 3B). Consistent with a defect in mitochondrial biogenesis, we found a reduction in the expression of nuclear genes encoding proteins involved in mitochondrial electron transport (cytochrome c and cytochrome oxidase IV) and oxidative phosphorylation (beta subunit of ATP synthase) in soleus muscle of PGC-1α^−/−^ mice compared with PGC-1α^+/+^ controls. In addition, the expression of Tfam, a known PGC-1α target involved in mitochondrial DNA replication/transcription, was diminished in PGC-1α^−/−^ soleus, providing one potential mechanism for defective mitochondrial biogenesis (Figure 3C). In contrast to the results with soleus, no significant differences in mitochondrial ultrastructure or volume density were noted in heart or BAT of PGC-1α^−/−^ mice (unpublished data).

To determine whether mitochondrial function was altered in the skeletal muscle of PGC-1α^−/−^ mice, mitochondrial respiration rates were measured using tissue strips prepared from soleus muscle. In soleus of PGC-1α^−/−^ mice, a significant defect in state 3 (ADP-stimulated) respiration, but not state 2 (basal), was detected using succinate as the substrate (Figure 3D). State 4 respiration rates (in the presence of oligomycin) were also similar between the genotypes, indicating that the coupling of respiration to ATP production was not significantly altered in PGC-1α^−/−^ mice. These results are consistent with the modest but significant reduction in mitochondrial volume density.

### Altered Skeletal Muscle Function in PGC-1α^−/−^ Mice

**Figure 4:**
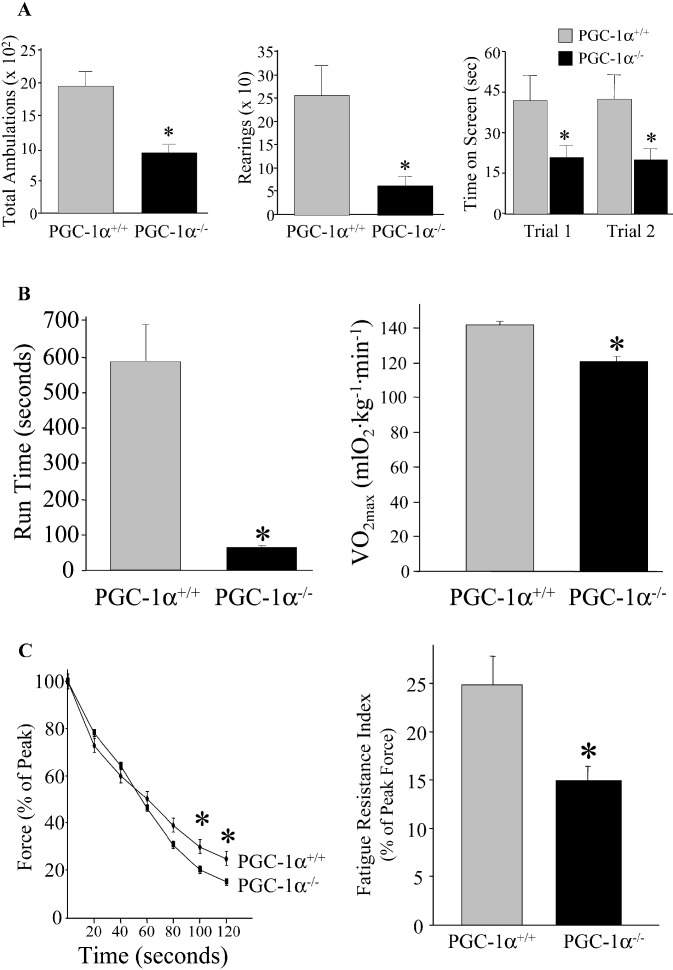
PGC-1α^−/−^ Mice Exhibit an Abnormal Skeletal Muscle Functional Phenotype (A) Measures of general activity and muscle strength. General activity levels were measured in 3.5-mo-old male PGC-1α^+/+^ (*n* = 8) and PGC-1α^−/−^ (*n* = 11) mice using a photobeam system as described in Materials and Methods. Total ambulations (left graph), and rearings (center graph) provide a general measure of locomotor activity. Time spent on an inverted screen (right graph) represents a general measure of extremity muscle strength. The results from two trials are shown. * *p* < 0.05 compared to corresponding PGC-1α^+/+^. (B) Exercise studies. Male 6–8-mo-old PGC-1α^−/−^ and PGC-1α^+/+^ mice were subjected to a run-to-exhaustion protocol on a motorized treadmill (left graph) as described in Materials and Methods. * *p* < 0.001 compared to corresponding PGC-1α^+/+^ values. VO_2max_ measurements were determined for 2-mo-old male mice for each genotype using a motorized treadmill at an elevation of 150 m and indirect calorimetry set-up (right graph) as described in Materials and Methods. * *p* < 0.05 compared to corresponding PGC-1α^+/+^ values. (C) Time course of fatigue following repeated stimulation of soleus muscle is shown for 4-mo-old male PGC-1α^−/−^ (*n* = 5) and PGC-1α^+/+^ (*n* = 5) mice (left graph). The mean percent force remaining at 2 min (Fatigue Resistance Index) is shown (right graph). * *p* < 0.05 compared to corresponding PGC-1α^+/+^ values.

The abnormality in mitochondrial number and respiratory function in skeletal muscle led us to further evaluate the skeletal muscle phenotype. As an initial step, we measured locomotor activity levels over a 1-h period using a high-resolution photobeam system. PGC-1α^−/−^ male mice exhibited a significantly lower mean number of ambulations and rearings during the hour compared to the PGC-1α^+/+^ age-matched controls (Figure 4). However, an analysis of exploratory behavior showed that the PGC-1α^−/−^ mice were reluctant to go into the center of the “field” compared to controls. Specifically, PGC-1α^−/−^ mice made significantly fewer entries into, spent significantly less time in, and traveled a significantly shorter distance in the central area of the “field,” although differences in distance traveled in the peripheral zone of the “field” was not significantly different between groups (Figure S2). These data suggest that the general activity level may have been affected by the reluctance of the PGC-1α^−/−^ mice to go into the central area of the field and thus remain in the periphery (thigmotaxis), possibly reflecting altered emotionality such as increased fear.

A battery of tests was performed to further evaluate the general sensorimotor phenotype of the PGC-1α^−/−^ mice. No differences were found between PGC-1α^−/−^ mice and PGC-1α^+/+^ controls on the ledge, platform, walking initiation, and 60° and 90° inclined screen tests (unpublished data), suggesting that several sensorimotor functions were intact in the PGC-1α^−/−^ mice. However, the PGC-1α^−/−^ mice were unable to remain on an inverted screen for as long as the PGC-1α^+/+^ controls (Figure 4A). Since the groups did not differ on the times it took to turn around and climb to the top of 60° and 90° inclined screens, the differences on the inverted screen test suggest that impaired strength rather than deficits in coordination were responsible for these differences.

To further evaluate the skeletal muscle phenotype, exercise capacity was assessed in the PGC-1α^−/−^ mice. To this end, the PGC-1α^−/−^ mice were exercised on a motorized treadmill apparatus using a run-to-exhaustion format. PGC-1α^−/−^ mice (6–8 mo of age) exhibited a markedly reduced capacity to sustain running exercise (PGC-1α^−/−^ mice, 64 ± 6 s; age-matched PGC-1α^+/+^ mice, 586 ± 104 s; Figure 4B). The same result was obtained with younger PGC-1α^−/−^ mice, i.e., at 3.5 mo of age (unpublished data). To quantify aerobic exercise capacity, VO_2max_ (maximum oxygen consumption, measured in milliliters of oxygen per kilogram of body weight per minute) was measured with the treadmill-running protocol using indirect calorimetry. VO_2max_ was significantly lower for the PGC-1α^−/−^ mice (120.9 ± 2.0 ml O_2_ · kg^−1^ · min^−1^) compared to PGC-1α^+/+^ controls (141.6 ± 2.1 ml O_2_ · kg^−1^ · min^−1^) (Figure 4B). To directly evaluate muscle fatigability, the force response to repetitive stimulation of isolated soleus muscle was determined. The capacity to generate force following a series of tetani is dependent upon mitochondrial ATP production. During the initial phase of the stimulation period, there was no difference in force generation in muscles isolated from PGC-1α^−/−^ mice and PGC-1α^+/+^ controls. However, fatigue resistance index, defined as the percent of initial force generated following a 2-min series of fatiguing contractions, was significantly lower in the PGC-1α^−/−^ mice (14.6 ± 1.5%) compared to PGC-1α^+/+^ controls (24.8 ± 2.9%) (Figure 4C). These results, together with the observed abnormalities in skeletal muscle mitochondrial structure and function, indicate that PGC-1α is necessary for functional adaptation of skeletal muscle to physiologic demands.

### Functional Abnormalities in Hearts of PGC-1α^−/−^ Mice

**Figure 5:**
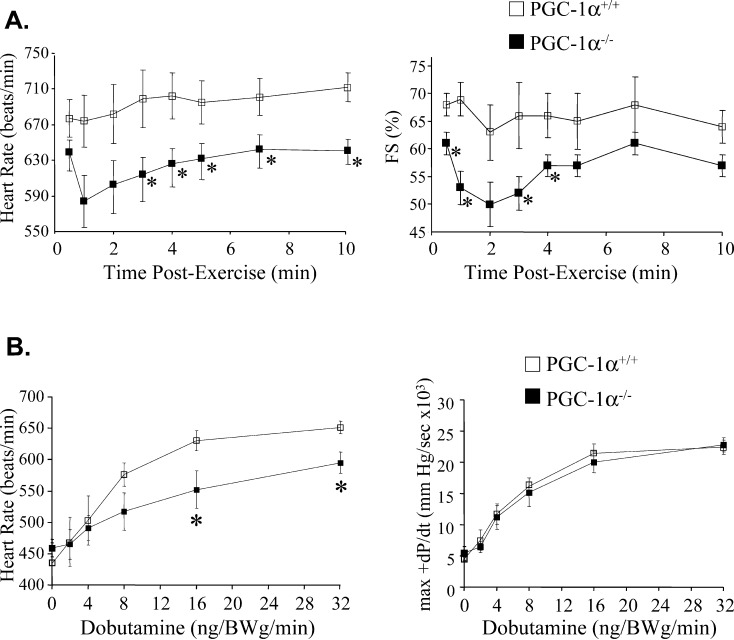
Abnormal Cardiac Response to Physiologic Stress in PGC-1α^−/−^ Mice (A) Exercise echocardiographic studies. PGC-1α^+/+^ (*n* = 4) and PGC-1α^−/−^ (*n* = 8) female mice aged 6–8 mo were subjected to an exercise protocol on a motorized treadmill. This protocol was designed such that the PGC-1α^−/−^ mice ran to exhaustion based on the results of the exercise studies shown in Figure 4. Accordingly, an exercise regimen of 60 s was used for both groups. The graphs depict the heart rate (left graph) and echocardiographically-determined ventricular fractional shortening (FS) as a percent (right graph). Responses were monitored for 10 min immediately post exercise. (B) In vivo hemodynamic response to the β_1_,α_1_-adrenergic agonist dobutamine. Male and female PGC-1α^+/+^ (*n* = 6) and PGC-1α^−/−^ (*n* = 6) mice at 10–12 wk of age were anesthetized and a 1.4-French Millar catheter was placed through the carotid artery into the left ventricle as described in Materials and Methods. Heart rate (left graph) and a measurement of ventricular systolic performance, dP/dt (right graph), were measured following infusion of dobutamine. * *p* < 0.05.

PGC-1α expression is enriched in heart, a tissue that relies heavily on mitochondrial energy metabolism to maintain pump function throughout the postnatal life of the mammalian organism. Echocardiographic screening studies of PGC-1α^−/−^ mice at ages 4–6 mo did not reveal any significant differences in chamber sizes or ventricular function compared to WT controls (unpublished data). Cardiac functional and metabolic reserve was evaluated using exercise echocardiographic stress testing (EST). Given that the exercise capacity of PGC-1α^−/−^ mice is diminished, a series of preliminary treadmill exercise studies were performed to define a reasonable exercise duration for run-to-exhaustion to be used as a target duration for the EST. Based on the results of these studies, an EST regimen was performed in which PGC-1α^+/+^ control animals were exercised for a duration of 60 s to match the predicted average for the PGC-1α^−/−^ mice (ages 6–8 mo). Echocardiographic images were obtained immediately following 60 s of exercise for the PGC-1α^+/+^ controls or at the point of exhaustion for PGC-1α^−/−^ mice (mean 60 ± 6.1 s, range 45–90 s). Echocardiographic-determined left ventricular fractional shortening and heart rate were monitored for the 10-min period immediately post exercise. The mean heart rate of the PGC-1α^−/−^ mice exhibited an inappropriate decline during the post exercise period (Figure 5A). In addition, echocardiographically determined left ventricular fractional shortening was decreased in the PGC-1α^−/−^ mice, but not the PGC-1α^+/+^ mice during the first 4 min of the post exercise period (Figure 5A).

The results of the EST did not distinguish between a primary cardiac abnormality versus effects secondary to the exhaustion caused by reduced exercise tolerance related to skeletal muscle dysfunction. To directly assess cardiac function, the hearts of PGC-1α^−/−^ and PGC-1α^+/+^ mice were isolated and perfused in the working mode. Hearts isolated from PGC-1α^−/−^ mice generated lower cardiac work (cardiac output multiplied by peak systolic pressure) compared to PGC-1α^+/+^ mice at identical loading conditions (Table 1). This reduction in cardiac work was due to a reduced cardiac output (Table 1). The relative contribution of heart rate and stroke volume to diminished cardiac output in the PGC-1α^−/−^ mice could not be delineated, because both were decreased but neither to a significant degree (Table 1). To further distinguish between abnormalities in heart rate and ventricular function, in vivo hemodynamic response to the β_1_,α_1_-adrenergic-selective agonist dobutamine was evaluated using a miniaturized Millar catheter. The ventricular functional response to dobutamine was similar in PGC-1α^+/+^ and PGC-1α^−/−^ mice (Figure 5B, right graph). However, PGC-1α^−/−^ mice exhibited a significantly blunted heart rate response to β-adrenergic stimulation (Figure 5B, left graph). Taken together with the EST, these results strongly suggest that the PGC-1α^−/−^ hearts are unable to mount an appropriate chronotropic response to exercise and other physiologic stimuli that activate β-adrenergic input to the heart. However, our results did not reveal evidence for contractile dysfunction.

**Table 1:**
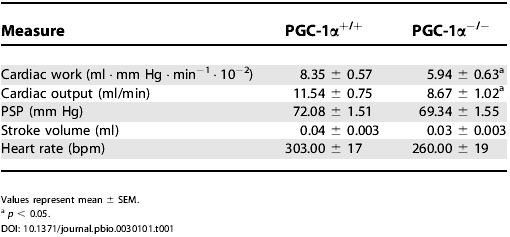
Cardiac Hemodynamics Measured in Isolated Working Hearts of PGC-1α^+/+^ and PGC-1α^−/−^ Mice

Values represent mean ± SEM

^a^ *p* < 0.05

### PGC-1α^−/−^ Mice Exhibit an Abnormal Thermogenic Response

**Figure 6:**
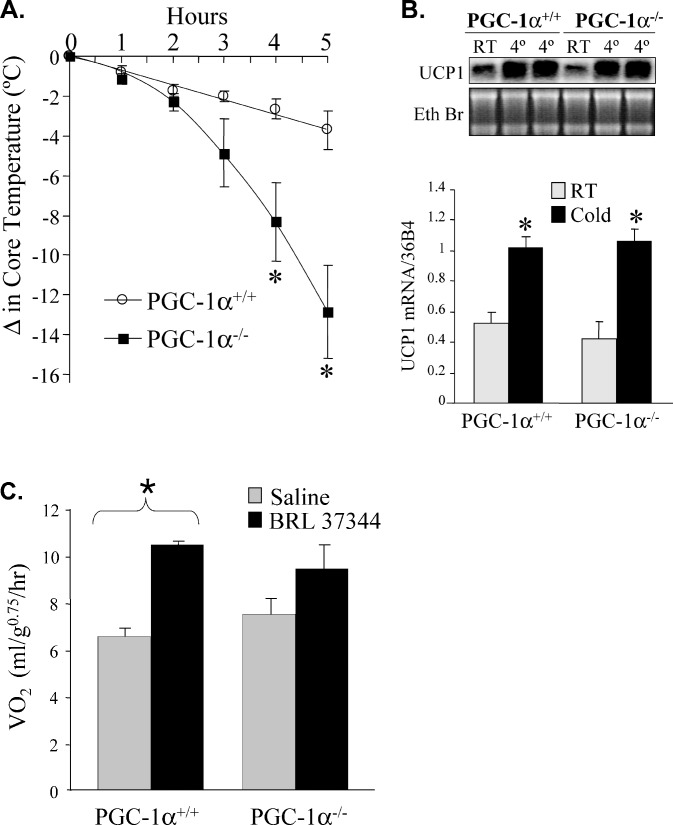
PGC-1α^−/−^ Mice Exhibit an Abnormal Thermogenic Response (A) PGC-1α^+/+^ (*n* = 15) and PGC-1α^−/−^ (*n* = 21) mice aged 28–37 d were subjected to cold (4 °C). Core rectal temperature was monitored over a 5-h period. The change in core temperature ± SEM is shown in the graph (left) as a function of time. * *p* < 0.05. (B) Representative Northern blot analysis (blot and gel at top) performed with RNA isolated from BAT to detect UCP-1 transcript at baseline (RT) and after 5 h of exposure to cold (4 °C) (UCP1). Ethidium bromide (Eth Br) staining of ribosomal RNA is shown as a control. Quantitative real-time RT-PCR for UCP-1 transcript is shown on the graph at the bottom. The values represent mean arbitrary units normalized to a 36B4 transcript (control). (C) Altered response to β_3_-adrenergic agonist. To evaluate the oxygen consumption (VO_2_) in response to the stimulation of BAT uncoupled respiration, the β_3_-adrenergic agonist BRL 37344 was administered to littermate PGC-1α^+/+^ (*n* = 5) and PGC-1α^−/−^ (*n* = 5) female mice followed by measurement of VO_2_ by indirect calorimetry. Mean ± SEM VO_2_ is shown. * *p* < 0.05.

PGC-1α has been implicated as an inducible regulator of mitochondrial respiratory uncoupling, an important source of heat production in BAT [2]. To determine whether PGC-1α is necessary for an appropriate thermogenic response, PGC-1α^+/+^ and PGC-1α^−/−^ mice were subjected to cold exposure (4 °C) for a 5-h period while core body temperature was monitored. PGC-1α^−/−^ mice exhibited a markedly abnormal drop in core temperature compared to the WT controls (Figure 6A). Specifically, the mean decline in core temperature was greater than 12 °C at the 5-h time point in PGC-1α^−/−^ mice, compared to an approximately 3 °C decrement in PGC-1α^+/+^ controls. Although this thermogenic phenotype was consistently present in mice aged 28–37 d, it was absent in older mice (unpublished data).

The histologic appearance and neutral lipid stores of BAT were assessed as an initial step to characterize the thermogenic phenotype exhibited by PGC-1α^−/−^ mice. Histologic and lipid quantification studies were performed. Electron microscopic analyses indicated that the mitochondrial ultrastructure was similar in BAT isolated from PGC-1α^−/−^ and PGC-1α^+/+^ mice before and after cold exposure (unpublished data). In addition, levels of BAT triglyceride were similar between the two genotypes (unpublished data). UCP-1 is a cold-inducible protein involved in mitochondrial respiratory uncoupling to generate heat in BAT. *UCP-1* gene transcription is known to be activated by PGC-1α [2]. Surprisingly, basal and cold-induced BAT UCP-1 mRNA levels were similar in PGC-1α^+/+^ and PGC-1α^−/−^ mice (Figure 6B). These results suggest that PGC-1α is not necessary for the induction of the expression of UCP-1 with cold exposure, and that other factors, such as reduced capacity for mitochondrial respiration, likely contribute to the abnormal thermogenic response in the PGC-1α^−/−^ mice.

Thermogenesis in rodents related to mitochondrial uncoupling is under the control of β_3_-adrenergic receptor coupled signaling. Accordingly, the in vivo oxygen consumption response to β_3_-adrenergic stimulation was examined in PGC-1α^−/−^ mice. For these experiments, VO_2_ (oxygen consumption) was measured following administration of the β_3_-agonist BRL 37344 using indirect calorimetry. VO_2_ was significantly increased in response to BRL 3744 in PGC-1α^+/+^ but not PGC-1α^−/−^ mice (Figure 6C). These results indicate that the metabolic response of BAT to an acute stimulus such as cold and/or β_3_-adrenergic stimulation is altered in the PGC-1α null mice, likely related to reduced capacity for mitochondrial respiratory uncoupling.

### Fasting-Induced Hepatic Steatosis in PGC-1α^−/−^ Mice

**Figure 7:**
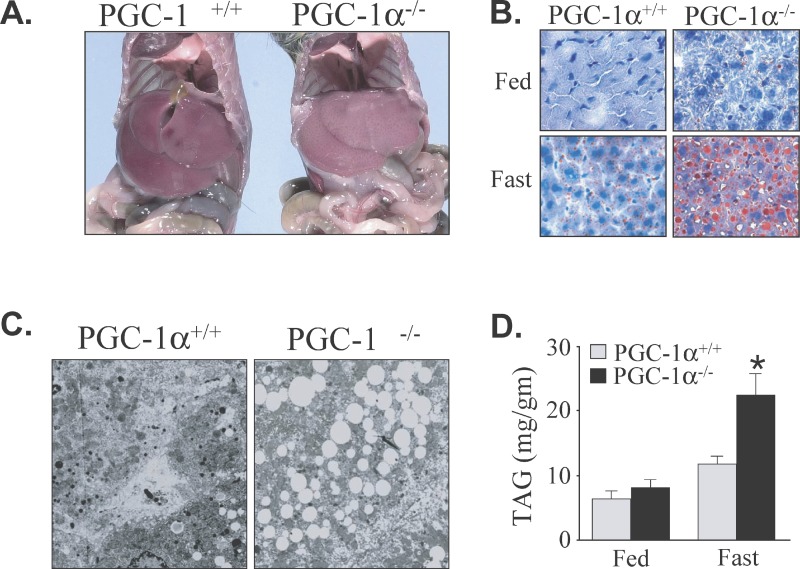
Fasting-Induced Hepatic Steatosis Develops in PGC-1α^−/−^ Mice (A) The photograph depicts the development of a pale liver in PGC-1α^−/−^ mice subjected to a 24-h fast. (B) Oil red O staining of histologic sections of liver taken from PGC-1α^−/−^ mice under fed and 24 h fasted conditions. The red staining indicates neutral lipid. (C) Representative electron micrographs of the liver from PGC-1α^+/+^ and PGC-1α^−/−^ mice following a 24-h fast. The droplets are indicative of neutral lipid accumulation. (D) Mean liver TAG levels in PGC-1α^+/+^ (*n* = 5) and PGC-1α^−/−^ (*n* = 5) mice under fed and 24-h fasted conditions. * *p* < 0.05.

**Figure 8:**
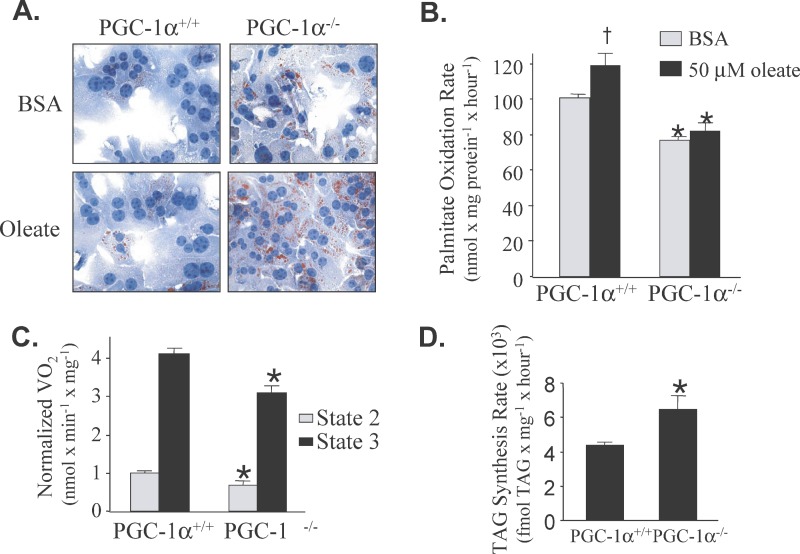
Hepatocytes Isolated from PGC-1α^−/−^ Mice Exhibit Reduced Oxidative Capacity (A) Oil red O staining of isolated hepatocytes exposed to BSA alone (BSA) or 50 μM oleate complexed to BSA (oleate). (B) ^3^H-palmitate oxidation rates. ^3^H-palmitate oxidation rates determined in hepatocytes isolated from PGC-1α^+/+^ and PGC-1α^−/−^ mice under cell culture conditions containing BSA or BSA + 50 μM oleate (2:1 oleate/BSA ratio). Values were derived from ten sets of triplicates for each group using hepatocytes from 5 mice of each genotype. The bars represent mean oxidation rates (*n* = 100) normalized to the condition of PGC-1α^+/+^ in BSA alone. * *p* < 0.05 compared to the corresponding PGC-1α^+/+^ mice. † *p* < 0.05 compared to PGC-1α^+/+^ with BSA treatment. (C) State 2 and 3 respiration rates determined for hepatocytes isolated from PGC-1α^+/+^ (*n* = 3) and PGC-1α^−/−^ (*n* = 3) mice using succinate/rotenone as a substrate. * *p* < 0.05 compared to corresponding PGC-1α^+/+^. (D) TAG synthesis rates in isolated hepatocytes. The bars represent mean TAG synthesis rates (glycerol incorporation, see Materials and Methods) for hepatocytes isolated from PGC-1α^+/+^ (*n* = 6) and PGC-1α^−/−^ (*n* = 6) mice. * *p* < 0.05 compared to the corresponding PGC-1α^+/+^ condition.

Previous studies have implicated PGC-1α in several hepatic metabolic functions including fatty acid oxidation and gluconeogenesis [18,19,20,21]. Accordingly, the hepatic phenotype was evaluated under basal conditions and following a 24-h fast, a stimulus known to induce fatty acid oxidation and gluconeogenic rates in liver. Under basal fed conditions, the livers of the PGC-1α^−/−^ mice appeared grossly normal and did not exhibit histologic abnormalities (unpublished data). However, following a 24 h-fast, the PGC-1α^−/−^ mice exhibited marked hepatic steatosis as determined by gross inspection, oil red O staining, electron microscopy, and measurements of liver triglyceride (TAG) levels (Figure 7). There were no differences in plasma triglycerides or free fatty acids between the genotypes in fed or fasted states (unpublished data). To further investigate the mechanisms involved in the fasting-induced hepatic steatotic response, hepatocytes were isolated from PGC-1α^−/−^ mice and WT controls. Oleate loading experiments revealed that the PGC-1α^−/−^ hepatocytes accumulated neutral lipid to a significantly greater extent than the WT cells (Figure 8A). ^3^H-palmitate oxidation rates were significantly lower in PGC-1α^−/−^ hepatocytes compared to PGC-1α^+/+^ hepatocytes under basal conditions and following exposure to oleate (Figure 8B). Taken together, these latter results indicate a cell-autonomous defect in PGC-1α^−/−^ hepatocytes that results in an inability to maintain cellular lipid balance in the context of increased delivery of lipid such as occurs with fasting.

PPAR, a known regulator of hepatic mitochondrial fatty acid oxidation enzyme gene expression, is a target for coactivation by PGC-1α [31]. Therefore, we sought to determine whether the steatotic phenotype of the PGC-1α^−/−^ mice related to reduced expression of PPAR target genes. To this end, a survey of candidate genes and gene expression profiling experiments were performed. Surprisingly, the hepatic expression of PPAR target genes involved in cellular fatty acid and oxidation (MCPT and MCAD) were not significantly different between the genotypes under fed or fasted conditions (Table 2). Next, we performed experiments to determine whether the reduced capacity for fat oxidation in the hepatocytes of the PGC-1α^−/−^ mice was related to altered mitochondrial respiratory function. Compared to the WT controls, PGC-1α^−/−^ hepatocytes exhibited a modest but significant reduction in both state 2 and state 3 respiration rates (Figure 8C). These results identify one potential mechanism responsible for the fasting-induced hepatic steatosis: reduced capacity for fat oxidation due to mitochondrial respiratory dysfunction.

**Table 2:**
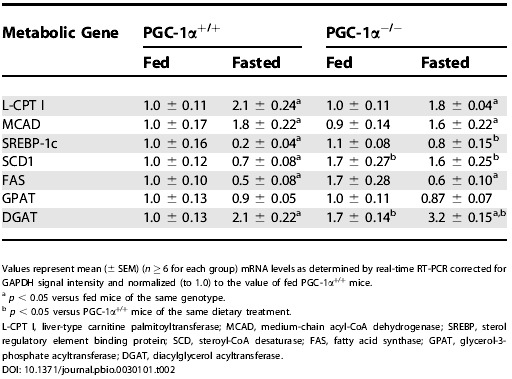
Metabolic Gene Expression in Liver of Fed and Fasted PGC-1α^+/+^ and PGC-1α^−/−^ Mice

Values represent mean (± SEM) (*n* ≥ 6 for each group) mRNA levels as determined by real-time RT-PCR corrected for GAPDH signal intensity and normalized (to 1.0) to the value of fed PGC-1α^+/+^ mice

^a^ *p* < 0.05 versus fed mice of the same genotype

^b^ *p* < 0.05 versus PGC-1α^+/+^ mice of the same dietary treatment

L-CPT I, liver-type carnitine palmitoyltransferase; MCAD, medium-chain acyl-CoA dehydrogenase; SREBP, sterol regulatory element binding protein; SCD, steroyl-CoA desaturase; FAS, fatty acid synthase; GPAT, glycerol-3-phosphate acyltransferase; DGAT, diacylglycerol acyltransferase

Although the liver gene expression profiling studies did not reveal abnormalities in the fatty acid oxidation pathway in the PGC-1α^−/−^ mice, several interesting differences in the activity of the sterol regulatory element binding protein-1c (SREBP-1c) pathway were noted. Specifically, the fasting-mediated down-regulation of SREBP-1c and its target gene stearoyl-CoA desaturase (SCD1), was abolished in PGC-1α^−/−^ mice (Table 2). Furthermore, expression of the gene encoding diglyceride acyltransferase (DGAT), which catalyzes the last step in TAG synthesis, was activated at baseline and induced by fasting to a greater level in PGC-1α^−/−^ mice (Table 2). These results suggest that, in addition to a defect in oxidation, components of the TAG synthesis pathway are activated in the PGC-1α^−/−^ mice. To evaluate this possibility directly, rates of ^3^H-glycerol incorporation into TAG were determined in isolated hepatocytes. ^3^H-TAG incorporation was increased nearly 50% in hepatocytes isolated from PGC-1α^−/−^ mice compared to PGC-1α^+/+^ controls (Figure 8D), confirming that TAG synthesis rates are increased in PGC-1α null hepatocytes, identifying a second potential mechanism contributing to the fasting-induced hepatic steatosis.

### Despite a Mild Obese Phenotype, Female PGC-1α^−/−^ Mice Do Not Exhibit Insulin Resistance

**Figure 9:**
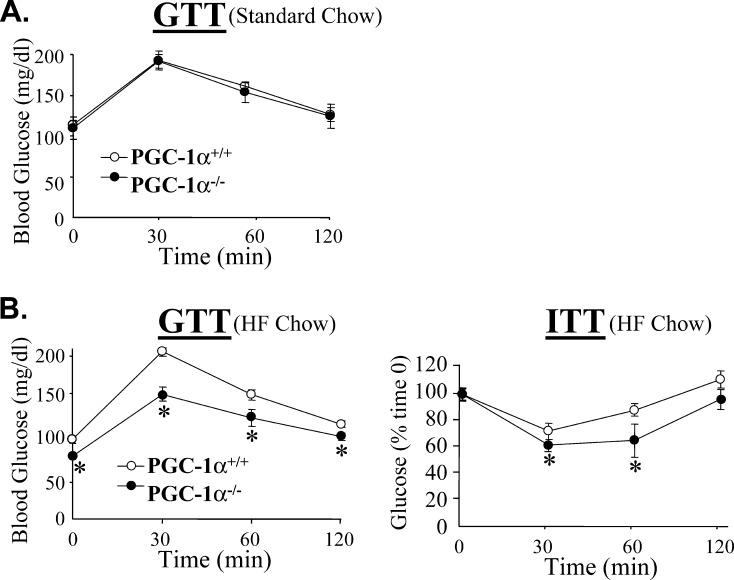
Female PGC-1α^−/−^ Mice Are More Glucose Tolerant and Insulin Sensitive Compared to PGC-1α^+/+^ on High-Fat Diet (A) At 4.5 mo of age, glucose tolerance testing (GTT) was performed on female PGC-1α^+/+^ (*n* = 6) and PGC-1α^−/−^ (*n* = 6) mice maintained on standard chow. (B) At 8 wk of age, PGC-1α^+/+^ (*n* = 8) and PGC-1α^−/−^ (*n* = 11) mice were provided a diet containing 43% of its calories from fat (HF chow). The graphs depict blood glucose levels ± SEM in PGC-1α^−/−^ mice during GTT (left graph) and ITT (right graph) studies. Studies were performed 5 wk (GTT) and 6 wk (ITT) after the initiation of the high-fat diet. * *p* < 0.05 compared to PGC-1α^+/+^ mice at the same time point.

Recent studies have suggested that specific *PGC-1α* single nucleotide polymorphisms and haplotypes may influence the development of insulin resistance and diabetes [27,30] and that PGC-1 activity is diminished in insulin-resistant and diabetic muscle [22,23]. Accordingly, peripheral glucose disposal and insulin responsiveness were examined in PGC-1α^−/−^ mice. Glucose tolerance testing of 2-mo-old male and female mice revealed no significant difference in blood glucose excursion between PGC-1α^+/+^ and PGC-1α^−/−^ groups (unpublished data). Given that older female PGC-1α^−/−^ mice develop an increase in body fat stores, glucose tolerance and insulin responsiveness were further evaluated in this group. Glucose tolerance testing in 4.5-mo-old female PGC-1α^−/−^ mice revealed that, despite increased body weight [mean ± standard error of the mean (SEM) weight of PGC-1α^+/+^ mice = 22.4 ± 0.79 g; PGC-1α^−/−^ mice = 25.2 ± 1.04 g), PGC-1α^−/−^ mice exhibited similar levels of glucose tolerance compared to WT mice on standard rodent chow (Figure 9). To examine glucose homeostasis in response to high-fat diet, female PGC-1α^+/+^ and PGC-1α^−/−^ mice were placed on high-fat chow (43% calories from fat) for 6 wk starting at 8 wk of age. The weight gained on the high-fat diet was similar for the PGC-1α^+/+^ and PGC-1α^−/−^ groups (Figure S3). Surprisingly, the PGC-1α^−/−^ mice on a high-fat diet were significantly more glucose-tolerant and insulin-sensitive compared to the PGC-1α^+/+^ mice (Figure 9B). Taken together, these results indicate that, despite excess body fat under standard conditions, the female PGC-1α^−/−^ mice do not exhibit insulin resistance. Moreover, the PGC-1α^−/−^ mice are more glucose-tolerant and insulin-sensitive than WT mice on a high-fat diet.

### Structural Abnormalities of the Central Nervous System in PGC-1α^−/−^ Mice

**Figure 10:**
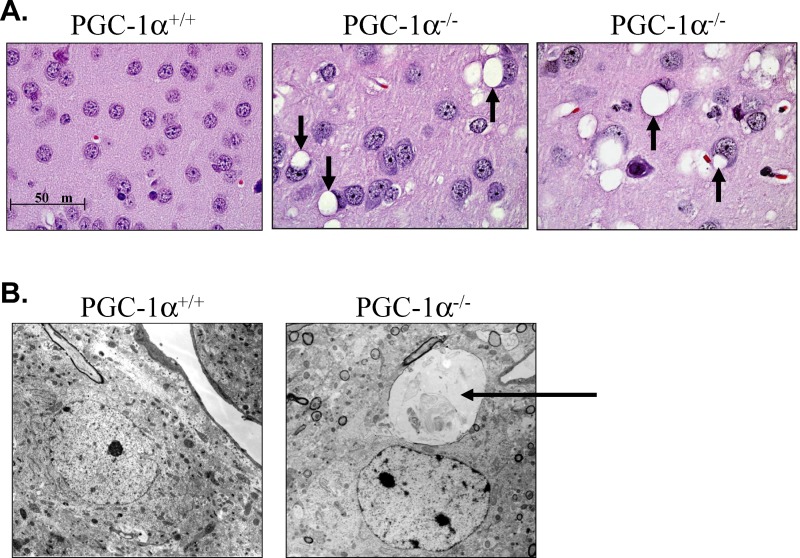
Neuropathology of the Central Nervous System of PGC-1α^−/−^ Mice (A) Light microscopic appearance of representative cerebral cortex of 2-mo-old PGC-1α^−/−^ mice demonstrates marked vacuolation of the neuropil (arrows) and scattered neuronal perikarya, which are absent in PGC-1α^+/+^ mice (hematoxylin and eosin). The scale bar shown is applicable to all sections. (B) Ultrastructural appearance of typical vacuoles containing membranous debris, denoted by the arrow, in the cerebral cortex of a representative PGC-1α^−/−^ mouse in comparison to PGC-1α^+/+^ (magnification 4000×).

In surveying the tissues of the PGC-1α^−/−^ mice, structural abnormalities of the brain were observed. Light microscopic examination of PGC-1α^−/−^ brain tissue samples demonstrated a well-preserved cerebral cortical neuronal complement, a result confirmed by measurement of neuron density in sections of the parietal lobe (PGC-1α^+/+^, 1,261 ± 91 neurons/mm^2^ versus PGC-1α^−/−^, 1,299 ± 82 neurons/mm^2^; not significant). Patchy areas of microvacuolation involving the neuropil and individual pyramidal neurons of the deep layers of the cerebral cortex were noted in the PGC-1α^−/−^ mice but not the PGC-1α^+/+^ mice (Figure 10A). Immunolocalization of an astrocytic marker, glial fibrillary acidic protein, failed to show an increase in numbers of astrocytic processes in PGC-1α^−/−^ mouse cerebral cortex (unpublished data). The hippocampus also showed neuronal microvacuolation, albeit to a lesser degree than the parietal cortex. Microvacuolation of the neuropil and neurons of the PGC-1α^−/−^ basal ganglia (caudate and putamen) was also noted in association with a patchy increase in the number and intensity of glial fibrillary acidic protein-immunoreactive astrocytic processes (unpublished data). Areas of microvacuolation also involved multiple brainstem regions. Only rare vacuolated Purkinje and granule cell neurons were identified in the PGC-1α^−/−^ cerebellar cortex. Neither microglial proliferation nor perivascular lymphocytic inflammatory infiltrates were noted in the PGC-1α^−/−^ CNS.

Ultrastructural examination of the PGC-1α^−/−^ parietal cerebral cortex confirmed the presence of microvacuolated neurons and neuropil (Figure 10B). Vacuoles containing aggregates of membranous material were present in a subset of cortical neurons. Subcellular localization of the vacuoles was difficult to establish; some may represent vacuolated elements of the neuropil, material in phagocytic cells, presynaptic nerve terminals compressing the soma, or genuine intraperikaryal deposits.

## Discussion

Previous studies using gain-of-function strategies have shown that the coactivator PGC-1α is capable of coactivating an array of transcription factors involved in energy metabolic processes including fatty acid oxidation, electron transport, and oxidative phosphorylation [6]. Forced expression of PGC-1α triggers mitochondrial biogenesis by activating a complex circuitry of factors including NRF-1, NRF-2, and the orphan nuclear receptor estrogen-related receptor α [23,32]. However, gain-of-function strategies cannot determine whether PGC-1α is essential for critical energy metabolic processes including mitochondrial biogenesis and function. Using targeted gene deletion in mice, we show here that PGC-1α is not essential for normal embryologic development or the fundamental events of mitochondrial biogenesis. However, several lines of evidence support the conclusion that PGC-1α is necessary for the programs that regulate postnatal mitochondrial function and cellular energy metabolism, processes that equip the organism for the energy metabolic rigors of the postnatal environment. First, mitochondrial volume density is diminished in slow-twitch skeletal muscle of PGC-1α^−/−^ mice. Second, mitochondrial respiratory capacity is modestly but significantly altered in skeletal muscle and liver of PGC-1α^−/−^ mice. Third, the growth of heart and soleus muscle, tissues with high reliance on mitochondrial energy production, is blunted. Fourth, control of body fat mass is abnormal in the PGC-1α^−/−^ mice. Finally, PGC-1α^−/−^ mice do not respond normally to a variety of physiologic and dietary stresses known to increase oxidative energy demands. Taken together, these results strongly suggest that PGC-1α is necessary for the terminal stages of mitochondrial maturation necessary to meet the energy demands of the postnatal environment.

Extensive phenotypic analyses demonstrated that mice lacking PGC-1α are unable to cope with physiologic stressors relevant to postnatal survival. For example, a skeletal muscle phenotype was unveiled in PGC-1α^−/−^ mice under conditions in which energy supply becomes limiting. This was most clearly demonstrated by the profound abnormalities exhibited by PGC-1α^−/−^ mice with exercise-to-exhaustion and repetitive muscle stimulation studies. Similarly, cardiac performance of PGC-1α^−/−^ mice was compromised following severe exertion. This effect was largely due to an abnormal heart rate response. The basis for the observed abnormalities of cardiac heart rate, including a blunted response to β-adrenergic stimulation, is unknown, but could be related to the effects of late-stage growth arrest and corresponding derangements in energy metabolism on sinus node function. PGC-1α was first identified as a coactivator in BAT [2]. Indeed, we found that exposure of the PGC-1α^−/−^ mice to cold, another relevant physiologic stress, resulted in an untoward drop in core body temperature consistent with an abnormality in thermogenesis despite normal cold induction of UCP-1 mRNA in BAT. Studies with a β_3_-adrenergic agonist confirmed that the peak oxygen consumption rate in thermogenic tissue is diminished in PGC-1α^−/−^ mice. We propose that the thermogenic phenotype is related to reduced capacity for mitochondrial respiration in BAT. Interestingly, this phenotype was only evident during a rather narrow window of postnatal life. Animals at an older age did not exhibit cold intolerance, possibly due to the insulating properties of increased body mass. Collectively, these results demonstrate the importance of PGC-1α as a key transducer of physiologic stimuli to the control of energy metabolism.

The observation of fasting-induced hepatic steatosis is another example of the inability of PGC-1α^−/−^ mice to respond to postnatal environmental metabolic demands. Following short-term starvation, we found that the PGC-1α^−/−^ mice developed marked hepatocyte triglyceride accumulation. Further analysis revealed that palmitate oxidation rates were reduced in hepatocytes isolated from the PGC-1α^−/−^ mice, which would predispose to lipid accumulation. Surprisingly, the reduction in fatty acid oxidation rates in PGC-1α null hepatocytes was not due to altered expression of PGC-1α/PPAR target genes involved in mitochondrial fatty acid oxidation. However, mitochondrial respiratory rates were diminished. In addition, we found that triglyceride synthesis was abnormally activated, and the expression of genes encoding SREBP-1c and SCD-1, key proteins in the hepatic lipogenic pathway, failed to be appropriately down-regulated in fasted PGC-1α^−/−^ mice. The mechanism involved in this latter finding is unknown. Indeed, the relative contribution of increased triglyceride synthesis rates to the steatotic phenotype cannot be fully discerned from our data, given that this response could reflect the direct effects of PGC-1α deficiency on target genes or a secondary compensatory response to hepatocyte fatty acid accumulation. Consistent with the former possibility, recent evidence indicates that PGC-1α coactivates the nuclear receptor FXR, a negative regulator of SREBP-1c expression and triglyceride synthesis [33]. We conclude that reduced hepatocyte mitochondrial respiratory capacity, and possibly activation of lipogenic programs, result in hepatocyte triglyceride accumulation in the context of increased hepatic delivery of fatty acids such as occurs with fasting.

We found that after 18 wk of age, female PGC-1α^−/−^ mice exhibit a mild but significantly abnormal weight increase associated with increased fat stores. Lean mass was unchanged at the time points examined. With further aging, a modest but significant increase in body fat was also noted in male PGC-1α^−/−^ mice (unpublished data). The basis for the observed abnormalities in weight control is unknown. We did not find differences in food intake or activity levels in female PGC-1α^−/−^ mice. It is possible that a reduction in systemic energy utilization, related to the mitochondrial dysfunction, leads to increased fat mass and weight gain in the PGC-1α^−/−^ mice. Interestingly, an association between *PGC-1α* gene polymorphisms and obesity in humans has been recently reported [26]. Clearly, future studies of male and female PGC-1α^−/−^ mice in pure-strain backgrounds over a range of ages will be necessary to fully investigate the observed abnormalities in weight control and fat distribution.

We did not find evidence for glucose intolerance or insulin resistance in the PGC-1α^−/−^ animals on standard chow. Moreover, female PGC-1α^−/−^ mice were more glucose-tolerant and insulin-sensitive than PGC-1α^+/+^ controls when consuming a high-fat diet. These findings are surprising, given the results of several recent studies demonstrating reduced expression of PGC-1α in human diabetic skeletal muscle [24,34]. It is certainly possible that compensatory metabolic regulatory mechanisms have been activated in the PGC-1α-deficient mice, accounting for this observation. Alternatively, PGC-1α could serve as a coactivator of factors that mediate diet-induced insulin resistance. Consistent with this notion, we and others have shown that mice lacking the PGC-1α target PPAR exhibit resistance to diet-induced glucose intolerance [21,35,36].

Histologic surveys of the PGC-1α^−/−^ mice revealed ultrastructural abnormalities in the central nervous system. Inspection of sections prepared from the brains of PGC-1α^−/−^ mice revealed patchy areas of microvacuolation in the pyramidal neurons of the cerebral cortex, accompanied by a mild increase in the number of astrocytes in the basal ganglia. The basis for this interesting but relatively nonspecific finding is unknown. It is possible that PGC-1α plays an important role in lipid metabolism related to membrane synthesis. Alternatively, the normal process of cellular debris turnover could be altered due to a defect in the energetics of the microglial component of the central nervous system. Although overt neurologic dysfunction was not apparent in PGC-1α^−/−^ mice during the first 6 mo of life (no group differences were found on five of six sensorimotor tests), the PGC-1α^−/−^ mice showed clear deficits on the inverted screen test. These deficits are likely due to impaired muscle strength in the PGC-1α^−/−^ mice, but contributions by peripheral or central nervous system determinants (or both) could be contributory. Moreover, evidence of altered emotionality from the 1-h locomotor activity test also suggests the possibility of altered brain function in PGC-1α^−/−^ mice. It will be of interest to determine whether the neurologic abnormalities contribute to the systemic metabolic abnormalities of the PGC-1α null mice.

During the preparation of this manuscript, Lin et al. reported an independent mouse line in which the PGC-1α gene was targeted [37]. Phenotypic comparison of the our PGC-1α-deficient line with that of Lin et al. reveals a number of similarities and several interesting differences. Both PGC-1α-deficient lines exhibit cold intolerance, reduced hepatocyte respiration rates, and neurologic lesions. However, a number of interesting differences are notable. First, in contrast to Lin et al., the PGC-1α^−/−^ mice described here do not exhibit any postnatal mortality. Second, we did not find evidence for a defect in gluconeogenesis based on fasting blood glucose levels (unpublished data). In addition, whereas Lin et al. found an abnormal expression profile for CCAAT-enhancer-binding protein β and δ and the gluconeogenic genes encoding phosphoenolpyruvate carboxykinase and glucose-6-phosphatase at baseline and with fasting in the PGC-1α^−/−^ mice, we did not (unpublished data). Third, we found evidence for an age-related increase in body fat in PGC-1α^−/−^ mice (females earlier than males), whereas Lin et al. identified a male-specific resistance to diet-induced obesity and insulin resistance. We have also found that male PGC-1α-deficient mice are somewhat protected against diet-induced obesity (Figure S4). However, we observed that the insulin-sensitive phenotype of the female PGC-1α^−/−^ mice occurred in the context of normal weight gain with high-fat diet. These latter results indicate that the insulin-sensitive phenotype of PGC-1α^−/−^ mice cannot be fully explained by a lean phenotype. Of interest, mice lacking the nuclear receptor estrogen-related receptor α, a known target of PGC-1α, exhibit resistance to diet-induced obesity similar to that of male PGC-1α null mice [38]. Fourth, the PGC-1α^−/−^ mice described here exhibit a dramatic fasting-induced hepatic steatotic phenotype, whereas the Lin et al. mouse does not. Fifth, Lin et al. found a neurologic phenotype in males characterized by hyperactivity, whereas the PGC-1α^−/−^ mice described here show reduced locomotor activity. However, it should be noted that we did not study activity levels over an extended period of time in males as did Lin et al., so it is possible that our findings reflect an emotional disturbance that manifests only when the animals are placed in a new environment. Finally, we report significant skeletal muscle and cardiac functional abnormalities (although the report by Lin et al. did not address these phenotypes, so this may not represent a true difference).

The reasons for the interesting differences between the two PGC-1α-deficient mouse lines are not clear. It is possible that distinct genetic backgrounds related to hybrid strains confer different degrees of secondary compensatory responses. In addition, the incompletely penetrant postnatal mortality noted in the PGC-1α^−/−^ mice reported by Lin et al. could have resulted in a selection bias toward greater levels of compensatory responses in liver and other tissues in the surviving group. It is also possible that the method of gene targeting led to different phenotypes. Lin et al. generated PGC-1α^−/−^ mice by Cre recombinase-mediated excision of exons 3–5 in oocytes. The PGC-1α^−/−^ mice described here were generated by a targeting event that involved a 3′ homologous recombination leading to an insertion of the targeting vector including an extra exon 3 between exons 5 and 6. The exon 3 insertion, which was confirmed by RT-PCR, results in a mutant transcript that encodes a truncated protein. We were unable to detect normal transcript containing an exon 5–6 border, indicating that the targeting was accurate and complete. In addition, we could not detect full-length or smaller PGC-1α proteins by Western blotting. However, we cannot exclude the possibility that the sensitivity of the immunoblotting was not high enough to pick up a small amount of mutant (truncated) PGC-1α protein that could have some activity, given that it would contain nuclear receptor-interacting domains and the amino-terminal activation domain. If small amounts of PGC-1α activity are present in the mice reported here, it could explain some of the observed differences between the models. However, the bulk of data presented here support the conclusion that the PGC-1α^−/−^ mice described are completely deficient in PGC-1α. Future direct comparison of the two mouse lines in pure background strains will be of interest.

In summary, this body of work provides evidence that PGC-lα is critical for the adaptive responses necessary to meet postnatal energy demands. Our results also suggest a broader role for inducible transcriptional coactivators such as PGC-1α in transducing cellular signals triggered by physiologic and developmental cues to the transcriptional control of energy metabolism and other dynamic cellular processes. In this regard, the inducible coactivator PGC-1α serves as a transcriptional “booster” to augment the capacity of downstream metabolic pathways critical for metabolic maturation and postnatal growth. Indeed, although PGC-1α null mice survive in the protected environment of the laboratory, our results indicate that in the rigors of a typical external environment, PGC-1α would be necessary for survival. Lastly, we propose that the PGC-1α^−/−^ mice should serve as a useful murine model to investigate the role of altered energy metabolism in obesity, diabetes, hepatic steatosis, and diseases of the heart, skeletal muscle, and central nervous system.

## Materials and Methods

### 

#### Targeting the PGC-1α gene in mice

A BAC genomic clone containing the murine *PGC-1α* gene, isolated from an Sv129 genomic library, was obtained from Incyte Genomics (Palo Alto, California, United States). A 3-kb region spanning exon 3 was amplified from the genomic clone. A 5′ primer was designed to amplify a fragment with the 5′ end beginning 732 bp upstream of exon 3 just upstream of an endogenous Kpn1 restriction site (5′- AGTTTCCTTAGCAACTTCATA-3′). The 3′ primer contained a BamH1 site engineered by mutating the bases shown in lowercase (5′- AAGGATTTTAgGATcc CAGTAC-3′). A second fragment downstream of exon 5 was amplified. In this latter amplicon, Not1 and Xho1 sites were engineered into the 5′ and 3′ primers, respectively (5′- TGGAGTgc GGCCGCTGGGA-3′ and 5′- AAAGAGTCTCgAg AATAGTTTCT-3′). The fragments were cloned into p1339-PGK-Neomycin targeting vector. The construct was linearized with Xho1 and electroporated into RW4 ES cells (Sv129 derived) using G418 selection. The electroporation was performed by the Siteman Cancer Center ES Cell Core at Washington University (St. Louis, Missouri, United States). The clones were screened by Southern blot using an Xba1 digest (see Figure 1A and 1B). One clone out of approximately 400 screened was positive for the homologous recombination on the 3′ end and an insertion on the 5′ end. This clone was injected into a C57BL6/J blastocyst. Chimeras were mated to C57BL6/J mice and germline transmission was confirmed by Southern blotting of tail DNA (see Figure 1B). All experiments were performed using sex- and age-matched or littermate controls as noted.

#### General animal studies

All animal experiments and euthanasia protocols were conducted in strict accordance with the National Institutes of Health guidelines for humane treatment of animals and were reviewed and approved by the Animal Care Committee of Washington University.

Animals were weighed at different time points. Male and female 3- to 8-wk-old PGC-1α^+/+^ and PGC-1α^−/−^ mice were euthanized, and tissues were dissected and weighed on an analytical balance. Tissue weights were corrected for total body weight before comparison. DEXA studies were performed as previously described [39] using a Lunar PIXIMUS DEXA system at 10, 18, and 24 wk in male and female PGC-1α^+/+^ and PGC-1α^−/−^ mice. For cold exposure experiments, male and female PGC-1α^+/+^ and PGC-1α^−/−^ mice were singly housed and placed at 4 °C for 5 h without food. Core body temperatures were monitored by rectal probe at baseline and every hour thereafter. Mice were monitored at least every 30 min to check for lethargy. At the end of 5 h, mice were sacrificed and tissues harvested for RNA and protein extraction. For fasting studies, animals were singly housed and given water ad libitum. Food was removed from cages in the morning and tissues harvested at 24 h for RNA and histology. Photography of the mice was performed by MedPic at Washington University School of Medicine. 48-h activity monitoring was performed by JAX Services (The Jackson Laboratory, West Sacramento, California, United States) using a Comprehensive Laboratory Animal Monitoring System (CLAMS, Columbus Instruments, Columbus, Ohio, United States). Briefly, 3-mo-old female mice were acclimated for 17 h before data collection. Data were collected every 30 min. Total beam breaks in the XY direction were tabulated for the 12-h light and dark cycles and compared across genotypes.

#### RNA, DNA, and protein analyses

Total RNA was isolated by the RNAzol method (Tel-Test, Friendswood, Texas) and Northern blotting was performed as previously described [40]. The PGC-1β and PRC cDNAs were generous gifts from Bruce Spiegelman and Richard Scarpulla, respectively. The UCP-1 cDNA was a gift from Daniel Ricquier. RT-PCR was performed as described [41]. In brief, total RNA isolated from soleus muscle, BAT, and heart of 1–2-mo-old PGC-1α^+/+^ or PGCα^−/−^ mice was reverse transcribed with Taqman reverse transcription reagents (Applied Biosystems, Foster City, California, United States). Reactions were performed in triplicate in 96-well format using Taqman core reagents and a Prism 7500 Sequence Detector (Applied Biosystems). The mouse-specific primer-probe sets used to detect specific gene expression can be found in Table S1. The primers for UCP-1 have been previously described [42]. Actin primer-probe set (Applied Biosystems) was included in a separate well and used to normalize the soleus, BAT, and heart gene expression data. GAPDH Rodent primers (Applied Biosystems) were used in the same well to normalize the liver gene expression data.

For Southern blot studies, 5 μg of genomic DNA was digested with Pst1 or Xba1, electrophoresed on a 0.8% TAE gel and transferred to a Gene Screen (Perkin Elmer, Wellesley, California, United States) membrane for hybridization. Western blotting was performed as described [43] using the Enhanced Chemiluminescence detection system (Amersham Pharmacia Biotech, Piscataway, New Jersey, United States). Ponceau S (Sigma, St. Louis, Missouri, United States) staining of the membrane was used as a control.

#### Mitochondrial respiration studies

Mitochondrial respiration was assessed in saponin-skinned soleus fibers with succinate as substrate and in the presence of rotenone as previously described [44]. In brief, 3-mo-old female mice were anesthetized with chloral hydrate (400 mg/kg of body weight). Soleus fibers were separated and then transferred to a buffer (2.77 mM K_2_Ca-EGTA, 7.23 mM K_2_EGTA, 6.56 mM MgCl_2_, 20 mM imidazole, 53.3 mM K-MES, 20 mM taurine, 5.3 mM ATP, 15 mM PCr, 3 mM KH_2_PO_4_, 0.5 mM DTT [pH 7.1]) supplemented with 50 μg/ml saponin and permeabilized for 30 min at 4 °C with gentle stirring. Fibers were then washed twice for 10 min each (2.77 mM K_2_Ca-EGTA, 7.23 mM K_2_EGTA, 1.38 mM MgCl_2_, 20 mM imidazole, 100 mM K-MES, 20 mM taurine, 3 mM KH_2_PO_4_, 0.5 mM DTT, 2 mg/ml BSA [pH 7.1]). Respiration was measured at 25 °C using an optical probe (Oxygen FOXY Probe, Ocean Optics, Dunedin, Florida, United States). Following measurement of basal state 2 respiration, maximal (ADP-stimulated) state 3 respiration was determined by exposing fibers to 1 mM ADP. The integrity of the outer mitochondrial membrane was assessed by adding 8 μM exogenous cytochrome c to ADP-stimulated fibers. State 4 respiration (uncoupled) was evaluated following addition of oligomycin (1 μg/ml). The solubility of oxygen in the respiration buffer at 25 °C was taken as 246.87 nmol O_2_ · ml^−1^. Respiration rates were expressed as nmol O_2_ · min^−1^ · mgdw^−1^.

#### Insulin and glucose tolerance tests

Glucose and Insulin tolerance tests were performed as described [35]. Prior to studies, mice were fasted overnight (GTT) or 6 h (ITT). In GTT studies, mice were injected with a 10% solution of D-glucose (1 g/kg). For ITT, mice received an IP injection of human regular insulin (Eli Lilly, Indianapolis, Indiana, United States) at a dose of 0.75 units/kg of body weight. Tail blood glucose was determined at 0, 30, 60, and 120 min after challenge using a B-GLUCOSE Analyzer (Hemacue AB, Angelholm, Sweden).

#### Indirect calorimetry

Oxygen consumption rates (VO_2_) of 5-wk-old female mice were measured using a Columbus Instruments Oxymax System. Resting baseline oxygen consumption rates were assessed for at least 1.0 h. For β_3_-adrenergic stimulation studies, BRL 37344 (Sigma) was dissolved in sterile saline and injected IP (2 μg/g of body weight) [45]. Postagonist assessment of oxygen consumption was recorded for an additional 1.0 h, with data collected at the 40-min time point.

#### Histology and electron microscopy

Soleus muscle and liver were dissected and fixed overnight in 2% glutaraldehyde, 1% paraformaldehyde, and 0.08% sodium cacodylate buffer. The tissues were postfixed in 1% osmium tetroxide, dehydrated in graded ethanol, embedded in Poly Bed plastic resin, and sectioned for electron microscopy. Cardiac and skeletal muscle mitochondrial and myofibrillar volume densities were determined from electron micrographs as described previously [10]. For each animal, three different fields at the magnification of 7500× were quantified in blinded fashion. Data were expressed as mean volume density of mitochondria or myofibrils in each field.

For electron microscopic analysis of the brain, tissue was prepared as previously described [46]. Ultrathin sections of cortex were cut onto formvar-coated slot grids stained with uranyl acetate and lead citrate and examined with a JEOL 1200 electron microscope. For H&E staining, sections of brain, including cerebral cortex, brainstem, and cerebellum, were dehydrated in graded concentrations of alcohol and embedded in paraffin from which 5-μm sections were prepared.

#### Primary mouse hepatocyte studies

Primary cultures of mouse hepatocytes were prepared from male PGC-1α^+/+^ and PGC-1α^−/−^ mice essentially as described [47]. Fatty acid oxidation and triglyceride synthesis experiments were commenced 2–3 h after the cells were plated. Triglyceride synthesis studies, were performed as previously described [47]. Palmitate oxidation rates were quantified using [9,10-^3^H]-palmitic acid as described [48] and corrected for total cellular protein content. For respiration studies, cells were spun down prior to plating and resuspended in a permeabilization buffer (described above) containing 50 μg/ml saponin. Respiration studies were performed in the presence of 5 mM succinate in the presence of 10 μM rotenone. Respiration rates were expressed as nmol O_2_ · min^−1^ · mg of protein^−1^.

#### Evaluation of locomotor activity, sensorimotor capabilities, and muscle function

To evaluate general activity levels and muscle use, mice were evaluated over a 1-h period in transparent (47.6 cm × 25.4 cm × 20.6 cm) polystyrene enclosures as previously described [49] using a high-resolution photobeam system (Motor Monitor, Hamilton-Kinder, Poway, California, United States). Each enclosure was surrounded by a frame containing a 4 × 8 matrix of photocell pairs, the output of which was fed to an on-line computer. The system software (Hamilton-Kinder) was used to define a 33 cm × 11 cm central zone and a peripheral or surrounding zone that was 5.5 cm wide with the sides of the cage being the outermost boundary. This peripheral area extended along the entire perimeter of the cage. Variables that were analyzed included the total number of ambulations, as well as the number of entries, the time spent, and the distance traveled in the center area as well as the distance traveled in the periphery surrounding the center. The total number of ambulations and rearings were recorded. For the inverted screen test, mice were placed on a wire mesh grid (16 squares per 10 cm) and the screen was inverted to 180°. A maximum score of 60 s was given if an animal did not fall.

The tests included in the sensorimotor battery [50] and accompanying protocols were designed as follows. (1) Inclined screen and inverted screen tests: For the inclined screen tests, each mouse was placed on top of an elevated (47 cm above the floor) wire mesh grid (16 squares per 10 cm) that was inclined to 60° or 90°. Each animal was placed in the middle of the screen with its head oriented down and was timed for how long it remained on the screen and how long it took to climb to the top of the screen. For the inverted screen test, mice were placed as above and then the screen was inverted to 180°. A maximum score of 60 s was given if an animal did not fall; (2) Platform test: Each mouse was timed for how long it remained on an elevated (47 cm above the floor) circular platform (1.0 cm thick and 3.0 cm in diameter). A maximum score of 60 s was assigned if the mouse remained on the platform for the maximum amount of time or if it could climb down on a very thin pole that supported the platform, without falling; (3) Ledge test: Each mouse was timed for how long it could maintain its balance on a 0.75-cm wide Plexiglas ledge without falling (60 s maximum). A score of 60 s was also assigned if the mouse traversed the entire length (51 cm) of the Plexiglas ledge and returned to the starting place in less than 60 s without falling; (4) Walking initiation test: Each mouse was placed in the middle of a square outlined by white cloth tape (21 cm × 21 cm) on a smooth black surface of a large tabletop. The time it took each mouse to leave the square (place all four paws outside of the tape) was recorded. The maximum time allowed was 60 s.

6–8-mo-old PGC-1α^+/+^ (*n* = 4) and PGC-1α^−/−^ (*n* = 8) mice were run to exhaustion employing a motorized, speed controlled, modular treadmill system (Columbus Instruments). The treadmill was equipped with an electric shock stimulus and an adjustable inclination angle. Running velocity was set at 35 m/min, with a level inclination angle.

#### VO_2max_ studies

VO_2max_ was determined while the mice were running on a treadmill using an open flow system (Columbus Instruments Oxymax System). All measurements of oxygen consumption took place at an elevation of 150 m (ambient P_BAR_ = 745 torr). Animals were placed into the metabolic chamber for 3–5 min to allow the system to equilibrate. Mice were then induced to run up an 18° incline at a speed of 40 m/min using a shock grid in the rear of the chamber. The speed was increased by 5 m/min every 2 min until the animals were unable to continue. Maximal effort was determined when oxygen uptake did not increase with power output and subsequently the mouse failed to maintain effort. VO_2max_ was calculated using the averaged values over 1 min during which the animal's O_2_ consumption reached a plateau.

#### Isolated muscle stimulation studies

Animals were anesthetized with ketamine/xylazine and the soleus muscle was removed from one leg. Upon removal, the muscle was suspended in a Krebs solution aerated with 95% O_2_ and 5% CO_2_. The muscle and Krebs solution were suspended within a water bath maintained at 37 °C, and the muscle was anchored to a Grass (West Warwick, Rhode Island, United States) isometric force transducer (model FTO3C). Muscles were stimulated to contract with a Grass stimulator (model S88) generating a field stimulus through electrodes located at both ends of the muscle. Force-voltage (maximal force at about 100 V) and length-tension relationships were determined using single twitch stimuli. The stimulator then delivered repeating trains of stimuli at one per second at 40 Hz for 2 min. Each train lasted 330 ms, and were digitally recorded using MacLab (AD Instruments, Colorado Springs, Colorado, United States). Fatigue resistance was calculated as the ratio of the force generated by the last tetanus divided by the highest force generated multiplied by 100 to give the percent of force generation that remained after the fatigue protocol.

#### Exercise echocardiography

Adult female mice (6–8 mo old) were exercised on the motorized treadmill using the run-to-exhaustion settings described above for 60 s or until exhaustion, whichever came first. Immediately following its treadmill run, the mouse was subjected to serial echocardiography using an Acuson Sequoia Echocardiography System performed as previously described [51].

#### In vivo cardiac hemodynamic studies

Hemodynamic studies were performed as previously described with some modifications [52]. In brief, adult mice (10–12 wk) were anesthetized intraperitoneally (IP) with thiopental sodium (60 mg/kg). The mice were intubated and ventilated with a Harvard ventilator. The right carotid artery was isolated in the region of the trachea and cannulated with a 1.4-French high-fidelity micromanometer catheter (Millar Instruments, Houston, Texas, United States), which was inserted into the left ventricle retrograde across the aortic valve. Hemodynamic measurements were recorded at baseline and 3 min following continuous infusion of incremental doses of dobutamine (β_1_, β_2_, and α_1_-adrenergic agonist) up to 32 ng · gBW^−1^· min^−1^ [53]. Continuous pressure-volume data were acquired and digitized with the BioBench computer software data acquisition system (National Instruments, Austin, Texas, United States).

#### Isolated working mouse heart perfusion

Isolated working mouse heart perfusion was based on a previously described procedure [54]. Adult mice (4–7 mo old) were heparinized (100 units IP) 10 min prior to anesthesia. Animals were then deeply anesthetized with 5–10 mg of sodium pentobarbital IP. Hearts were excised and placed in an ice-cold Krebs-Henseleit bicarbonate (KHB) solution [118 mM NaCl, 25 mM NaHCO_3_, 4.7 mM KCl, 1.2 mM KH_2_PO_4_, 2.5 mM CaCl_2_, 5.0 mM glucose, and 100 units/L insulin (pH 7.4)]. Hearts were cannulated first via the aorta and perfused retrogradely by the Langendorff method. Following left atrial cannulation, perfusion was switched to the working mode with KHB solution containing 1.2 mM palmitate bound to 3% fatty acid-free BSA with a preload pressure of 11.5 mm Hg and an afterload pressure of 50 mm Hg for 60 min with oxygenated buffer solution. Functional measurements, namely cardiac output, aortic flows, peak systolic pressure, and heart rate were acquired every 10 min using inline flow probes (Transonic Systems, Ithaca, New York, United States), a pressure transducer (TSD 104A, BIOPAC Systems, Santa Barbara, California, United States) and data acquired with the MP100 system from AcqKnowledge (BIOPAC Systems). Cardiac work was calculated as the product of peak systolic pressure and cardiac output.

#### Statistics

Data were analyzed using T-tests or ANOVAs (measures of general activity and sensorimotor battery). The level of significance was set at *p* < 0.05 in all cases. Data are reported as mean values ± SEM, unless otherwise noted. The ANOVA model used to analyze each sensorimotor test included one between-subjects variable (genotype), and one within-subjects variable (trials). When ANOVAs with repeated measures were conducted, the Huynh-Feldt (H-F) adjustment of alpha levels was used for all within-subjects effects containing more than two levels, in order to protect against violations of the sphericity/compound symmetry assumptions underlying this ANOVA model. In addition, Bonferroni correction was used when appropriate to help maintain prescribed alpha levels (e.g., *p* < 0.05) when multiple comparisons were conducted.

## Supporting Information

Figure S1 Activity Levels in Female PGC-1α^−/−^ Mice Is Unchanged Using a CLAMS system, PGC-1α^+/+^ (*n* = 4) and PGC-1α^−/−^ (*n* = 3) female mice were monitored for 48 h after a 17-h period of acclimation. XY beam breaks were tabulated over the 12-h light and dark cycles as denoted on the bottom. The bars represent mean (± SEM) beam breaks per each 12-h cycle.
(342 KB EPS).

Figure S2 Altered Emotionality in PGC-1α^−/−^ Mice An analysis of exploratory behavior included the number of entries into the center of the cage (upper left), the time spent in the center of the cage in seconds (sec) (upper right), the distance traveled in the center of the cage in meters (m) (lower left) as well as the distance traveled in the periphery (lower right). * *p* < 0.05 compared to the PGC-1α^+/+^ mice.
(620 KB EPS).

Figure S3 No Difference in Weight Gain on a High-Fat Diet in Female PGC-1α^−/−^ Mice Compared to WT Controls 8-wk-old female mice were fed a diet high in fat (43% calories from fat) for 6 wk. The change in body weight (grams) after 6 wk on a high-fat diet is shown for PGC-1α^+/+^ (*n* = 8) and PGC-1α^−/−^ (*n* = 11) mice. NS, not significant.
(264 KB EPS).

Figure S4 Male PGC-1α^−/−^ Mice Are Somewhat Resistant to Diet-Induced Obesity Male and female PGC-1α^+/+^ (*n* ≥ 6) and PGC-1α^−/−^ (*n* ≥ 6) mice were fed a high-fat diet (43% calories from fat) beginning at 4 wk of age. Body weight was monitored weekly as shown on the graph on the left. The mean (± SEM) change in body weight is shown in the bar graph on the right. *, significant difference compared to the PGC-1α^+/+^ controls, *p* < 0.05.
(794 KB EPS).

Table S1 Probes and Primers Sequences of mouse-specific probes and primers used for real-time RT-PCR.
(25 KB DOC).

### Accession Numbers

The GenBank (http://www.ncbi.nlm.nih.gov/) accession numbers of the vector discussed in this paper is p1339-PGK-Neomycin targeting vector (AF335420).

## Abbreviations

BAT: brown adipose tissue
DEXA: dual-energy X-ray absorption
EST: echocardiographic stress testing
IP: intraperitoneal(ly)
NRF: nuclear respiratory factor
PGC: PPARγ coactivator
PPAR: peroxisome proliferator-activated receptor
PRC: PGC-1 related coactivator
SCD: steroyl CoA desaturase
SREBP: sterol regulatory element binding protein
SEM: standard error of the mean
TAG: triglyceride
Tfam: mitochondrial transcription factor A
UCP: uncoupling protein
WT: wild-type
