# Supplementary material for: PGC-1α Deficiency Causes Multi-System Energy Metabolic Derangements: Muscle Dysfunction, Abnormal Weight Control and Hepatic Steatosis
Source: PLoS Biol. 2005 Mar 15;3(4):e101. doi: 10.1371/journal.pbio.0030101 (PMC1064854; doi:10.1371/journal.pbio.0030101)
Supplement: Supplementary file 1 [file pbio.0030101.st001.doc]

# Supplementary Table 1

| **Gene** | **Forward Primer** | **Reverse Primer** | **Probe** |  |
| --- | --- | --- | --- | --- |
| Cytochrome C | ACCAAATCTCCACGGTCTGTT | GGATTCTCCAAATACTCCATCAG | ACAGATGCCAACAAGAACAAAGGCAT | FAM |
| ATP Synthase β | CGTGAGGGCAATGATTTATACCAT | TCCTGGTCTCTGAAGTATTCAGCAA | ACCAACGCTACCTTGGAAGTGGCATCT | FAM |
| Tfam | AGGCTTGGAAAAATCTGTCTC | TGCTCTTCCCAAGACTTCATT | AAAGCAGGCATATATTCAGCTTGCT | FAM |
| COXIV | TACTTCGGTGTGCCTTCGA | TGACATGGGCCACATCAG | TAGTCTTCACTCTTCACAACACTCCCATGT | FAM |
| UCP-1 | CGACTCAGTCCAAGAGTACTTCTCTTC | GCCGGCTGAGATCTTGTTTC |  | SYBR |
| PGC1α Exon 5-6 | AACCGCAGTCGCAACATG | GTTCGCTCAATAGTCTTGTTCTCA | TCAAGCCAAACCAACAACTTTATCTCTTCC | FAM |
| PGC1α Exon 5-3 | ATACACAACCGCAGTCGCAACA | TCTGTGAGGACCGCTAGCAAGTTT |  | SYBR |
| PGC1α Exon 10-11 | AGAGAGGCAGAAGCAGAAAGCAAT | ATTCTGTCCGCGTTGTGTCAGG |  | SYBR |

**Sequence of mouse-specific probes and primers used for real-time RT-PCR.**
